# A BAC library of the SP80-3280 sugarcane variety (*saccharum* sp.) and its inferred microsynteny with the sorghum genome

**DOI:** 10.1186/1756-0500-5-185

**Published:** 2012-04-23

**Authors:** Thais Rezende e Silva Figueira, Vagner Okura, Felipe Rodrigues da Silva, Marcio Jose da Silva, Dave Kudrna, Jetty SS Ammiraju, Jayson Talag, Rod Wing, Paulo Arruda

**Affiliations:** 1 Centro de Biologia Molecular e Engenharia Genética, Universidade Estadual de Campinas (UNICAMP), Campinas, SP, 13083-875, Brazil; 2 EMBRAPA Informática na Agricultura, São Paulo, Brazil; 3 Arizona Genomics Institute, School of Plant Sciences, BIO5 Institute, University of Arizona, Tucson, AZ, 85721, USA; 4 Departamento de Genética e Evolução, Instituto de Biologia, Universidade Estadual de Campinas (UNICAMP), Campinas, SP, 13083-875, Brazil

**Keywords:** Sugarcane genomics, BAC library, Genome organization, Microsynteny, Sorghum

## Abstract

**Background:**

Sugarcane breeding has significantly progressed in the last 30 years, but achieving additional yield gains has been difficult because of the constraints imposed by the complex ploidy of this crop. Sugarcane cultivars are interspecific hybrids between *Saccharum officinarum* and *Saccharum spontaneum*. *S. officinarum* is an octoploid with 2n = 80 chromosomes while *S. spontaneum* has 2n = 40 to 128 chromosomes and ploidy varying from 5 to 16. The hybrid genome is composed of 70-80% *S. officinaram* and 5-20% S*. spontaneum* chromosomes and a small proportion of recombinants. Sequencing the genome of this complex crop may help identify useful genes, either per se or through comparative genomics using closely related grasses. The construction and sequencing of a bacterial artificial chromosome (BAC) library of an elite commercial variety of sugarcane could help assembly the sugarcane genome.

**Results:**

A BAC library designated SS_SBa was constructed with DNA isolated from the commercial sugarcane variety SP80-3280. The library contains 36,864 clones with an average insert size of 125 Kb, 88% of which has inserts larger than 90 Kb. Based on the estimated genome size of 760–930 Mb, the library exhibits 5–6 times coverage the monoploid sugarcane genome. Bidirectional BAC end sequencing (BESs) from a random sample of 192 BAC clones sampled genes and repetitive elements of the sugarcane genome. Forty-five per cent of the total BES nucleotides represents repetitive elements, 83% of which belonging to LTR retrotransposons. Alignment of BESs corresponding to 42 BACs to the genome sequence of the 10 sorghum chromosomes revealed regions of microsynteny, with expansions and contractions of sorghum genome regions relative to the sugarcane BAC clones. In general, the sampled sorghum genome regions presented an average 29% expansion in relation to the sugarcane syntenic BACs.

**Conclusion:**

The SS_SBa BAC library represents a new resource for sugarcane genome sequencing. An analysis of insert size, genome coverage and orthologous alignment with the sorghum genome revealed that the library presents whole genome coverage. The comparison of syntenic regions of the sorghum genome to 42 SS_SBa BES pairs revealed that the sorghum genome is expanded in relation to the sugarcane genome.

## Background

Sugarcane is a C4 plant that stores 1/3 of its fixed carbon as sucrose in the parenchyma cells of mature stalks. The other 2/3 is stored in the leaves (1/3) and, the stalks (1/3) in the form of complex carbohydrates [1]. Sugarcane has been grown as a sugar source for a century, but in recent years, extensive industrial plantations have demonstrated this crop’s value for the production of sustainable energy [2]. In industrial plantations, when sugarcane is harvested, its leaves are left in the field, contributing to the improvement of soil conservation and fertility. The stalks are transported to sugarcane mills and crushed. After crushing the juice enters a first-pass sucrose crystallisation, and the sugar remaining in the molasses goes to fermenters to produce fuel ethanol [3]. Currently, the dried bagasse resulting from the stalk crushing is used to produce bioelectricity, but it could also be used for the production of cellulosic ethanol [1]. Sugarcane juice has also been used as a carbon source by the synthetic biology industry to produce other fuels and high value molecules [3]. However, the worldwide use of sugarcane for sustainable energy production depends, on the development of superior varieties that are able to grow in less fertile soils, in stress-inducing biotic and abiotic conditions in a range of tropical and sub-tropical environments.

The cultivated sugarcane varieties derive from crosses performed at the beginning of the last century between *S. officinarum*, a species with a high sugar content in the stalk and *S. spontaneum*, a disease-resistant and vigorous wild relative [4,5]. After few backcrosses of the interspecific hybrid to *S. officinarum*, the breeders were able to select varieties less sensitive to biotic and abiotic stress and with a high sugar content in their stalks [5,6]. These early interspecific hybrids constitute the basic germplasm used in breeding programs around the world. However, breeding sugarcane is a complex task because of the high degree of ploidy of the ancestor species [7,8]. *S. officinarum* is octoploid with a basic chromosome number of x = 10 and 2n = 80 chromosomes, while *S. spontaneum* has a basic chromosome number of x = 8 and 2n = 40 to 128, and a ploidy varying from 5 to 16 [9,10]. The interspecific hybrid genome is a mixture of the genomes of both species with a ploidy varying between 2n = 100 and 2n = 130 chromosomes [11]. Intact chromosomes from both parents coexist in the interspecific hybrid in proportion of 5-20% from *S. spontaneum* and 70-80% from *S. officinarum*, along with a variable proportion of recombinants between the parental homoeologous chromosomes [12]. This genome architecture imposes constraints for the breeding process and prevents the use of seeds for progeny propagation because of the complex allelic segregation from the polyploidy hybrid [2]. This has limited the achievement of genetic gains in breeding programs, despite the use of crosses between numerous selected parental varieties and evaluation of hundreds of thousands or even millions of progenies in the large-scale field trials.

Because of its complexity, the complete sugarcane genome has not yet been sequenced, mainly due to the difficulty of assigning gene-containing fragments to a specific homologous/homeologous chromosome. However, a reference genome sequence could be assembled from fragments of different homologous and homeologous chromosomes and, even though this reference sequence would be chimeric, it could be useful for comparative genome analysis with close relatives, such as sorghum [13].

The estimated monoploid genome size of sugarcane is approximately 760–930 Mb [7], which is close to the 730 Mb size observed for sorghum [14]. A reference sugarcane genome sequence can be obtained by sequencing a representative bacterial artificial chromosome (BAC) library. Few sequenced BAC clones from the commercial Reunion Island R570 sugarcane variety has already demonstrated the viability of comparative genomics between sugarcane and sorghum [15-17].

This report describes the construction and initial analysis of a BAC library from the Brazilian sugarcane variety SP80-3280, which has been extensively cultivated during the past 18 years [2]. This library will be made available for the scientific community, and would be useful for the establishment of a reference genome sequence for sugarcane. The library was characterised in terms of insert size and genome coverage based on the alignment of a random sample of BAC end sequences (BESs) into the sorghum genome. Gene annotation of these BESs provided an early glimpse into the sequence composition of the sugarcane genome compared to the sorghum genome.

## Results

### Construction and characterisation of the SP80-3280 BAC library

The sugarcane variety SP80-3280 was chosen to construct the BAC library because it has been widely cultivated in Brazil. Around 300 thousand Ha has been cultivated with SP80-3280 along the past, recent years in different regions of the country. The superior agronomic performance in such a vast area implies that breeders have selected adaptability traits responsible for yield stability. Thus, sequencing a BAC library from this variety may reveal allelic composition involved in crop performance, and by comparing with genome sequence from other sugarcane BAC libraries may reveal genomic regions responsible for crop adaptation to different environments. The SP80-3280 has also contributed to the cDNA libraries used for EST sequencing carried out by the sugarcane EST project (SUCEST) [18]. SUCEST sequences targeted over 70% of the expressed sugarcane genes [19] and have demonstrated its usefulness for genome annotation of sugarcane BAC sequences [17].

**Figure 1:**
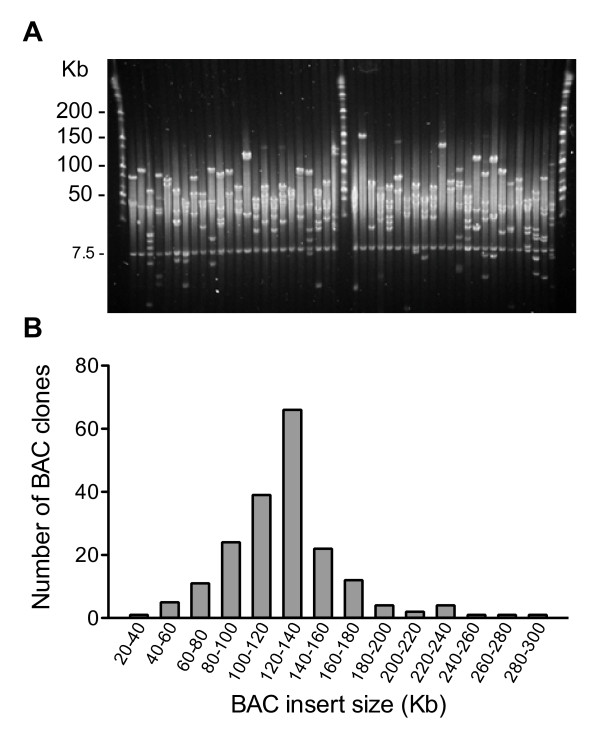
**Insert size distribution in a random sample of 192 BAC clones of the SP80-3280 sugarcane BAC library.** (**A**) Example of pulsed-field gel electrophoresis (PGE) of 48 BAC clones DNA digested with *Not*I. Lanes 1, 26 and 51 are Lambda Ladder PFG (New England Biolabs) molecular weight DNA markers. The 7.5-Kb band marks the position of the *Not*I-released cloning vector. (**B**) Insert size distribution of the 192 BAC clones as estimated by *Not*I digestion and PGE.

High molecular weight (HMW) genomic DNA prepared from the isolated nuclei of young sugarcane leaves was partially digested with *Hind*III, and the fragments were fractionated by pulsed-field agarose gel electrophoresis [20]. After elution from the gel, the fragments were ligated into the *Hind*III cloning site of the pAGIBAC1 vector, and the ligations were transformed into the DH10B T1 *E. coli* strain to generate the SS_SBa BAC library comprised of 36,864 BAC clones (Table 1). Based on a genome size of 760–930 Mb for the monoploid chromosome set [7], we estimated that this library corresponds to approximately 5-6-monoploid sugarcane genome equivalents. However, as has recently been suggested based on the sequences of 19 BACs from the R570 sugarcane variety [17], the sugarcane genome could be 20% smaller than that of sorghum; therefore, the SS_SBa BAC library could represent 8-fold coverage of the monoploid sugarcane genome. The library was picked into 96 x 384-well plates, and 192 BAC clones, two for each 384-well plate, were randomly selected for insert size estimation and BAC end sequencing. *Not*I restriction enzyme digestion showed that the library was composed of large insert clones (Figure 1A) with an average estimated insert size of 125 Kb (ranging from 29 to 293 Kb), 87.5% of which contained inserts larger than 90 Kb (Figure 1B). Restriction analysis of this 192 BAC clone sample revealed an absence of empty vectors among the 36,864 clones of the SS_SBa BAC library. The 36,864 SS_SBa BAC library clones were printed onto hybridisation screening filters for future experiments.

**Table 1:** Summary of the SS_SBa Sugarcane BAC library

**Germplasm Cloning vector**	**Sugarcane variety SP80-3280 pAGIBAC1**
Partial digest enzyme	*HindIII*
Number of clones	36,864
Number of 384-well plates	96
Number of analyzed clones	192
Average insert size (kb)	125
Minimum insert size (kb)	29
Maximum insert size (kb)	293
Number of high quality BES	378
Average BES read length (bp)*	944
Chloroplast contamination (%)	0.5
Mitochondrial contamination	None
Number of monoploid genome equivalents**	5-6 X

### BES of a clone sample of the SS_SBa BAC library

The quality of the library and its potential genome coverage were examined by bidirectional end sequencing of the randomly selected 192 BAC clones for insert size estimation and its alignment to the genome sequence of the 10 sorghum chromosomes (Additional file 1: Table S1). After trimming the BES sequence reads for low quality and vector bases, 378 sequences, with an average read length of 944 nucleotides and a minimum length of 312 bases, were recovered (Table 1).

**Figure 2:**
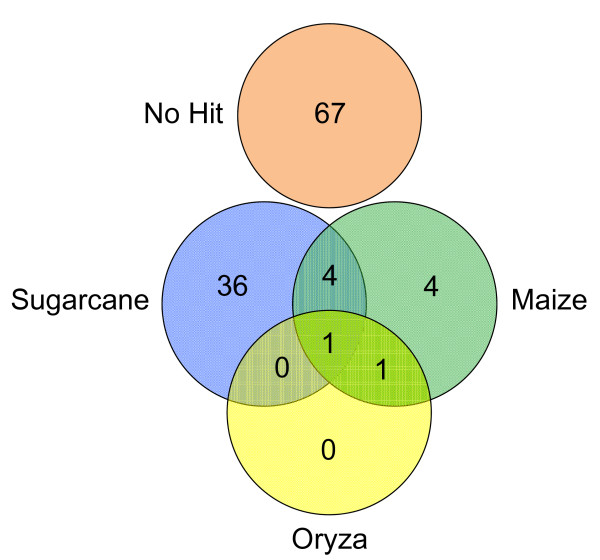
Distribution of BlastN hits among maize, rice and sugarcane of the 118 BES for which no significant hits against the sorghum genome were obtained.

The sugarcane BESs were compared to the sugarcane chloroplast genome [21] and the rice mitochondria genome [22]. No significant similarity to mitochondrial genome was found in the library while 1 BAC, out of 192 (0.5%), showed similarities with chloroplast genome (Table 1). Among the 378 BES sequences, 113 produced no hits with sorghum, either at the nucleotide or protein sequence level. Of these 113 sequences, 67 produced no significant hit against any nucleotide or protein sequence in GenBank, and 36 produced significant hits exclusively with sugarcane (Figure 2). These 103 BES with no hit with the sorghum genome may represent sugarcane-exclusive sequences. This result is in keeping with those observed by the analysis of 19 sugarcane BAC sequences from the R570 sugarcane variety BAC library [17] and analysis of the sugarcane ESTs [19]. Among the remaining 10 BES with no hit against sorghum, 4 produced significant hits exclusively with maize, 4 with maize and sugarcane, 1 with maize and rice and 1 with maize, rice and sugarcane (Figure 2). These BESs may represent conserved sequences from the Andropogoneae ancestor that gave rise to grasses but, may have been lost by the sorghum genome after the divergence of Saccharum/sorghum that occurred approximately 8 Ma ago (MYA) [7,16,17].

### Synteny and micro-collinearity with sorghum

The 378 BESs were aligned with the 10 sorghum chromosomes in search for synteny and micro-collinearity. From the 265 positive alignments, 84 BESs, corresponding to the end sequence pairs of 42 BACs (Table 2, Class1), aligned in a concordant manner with the genome sequence of at least one of the 10 sorghum chromosomes, indicating conformity to sugarcane/sorghum syntenic genome regions. This BES category was assigned as Class 1 and comprises all concordant alignments. Another set of 88 BESs, corresponding to 44 BACs, had both BES end aligned to genome sequences of the same sorghum chromosomes (Table 2, Classes 2 to 5). However, their BES sequence pairs aligned in a discordant manner - in the same orientation or at positions smaller than 20 Kb or larger than 300 Kb. These sequences may correspond to sugarcane genome regions that were inverted, expanded or contracted after the divergence of sugarcane/sorghum. A set of 18 BES, corresponding to 9 BACs, presented end sequence pairs aligned with different sorghum chromosomes (Table 2, Class 6). These sequences may represent sugarcane regions that were rearranged by translocation after the sugarcane/sorghum divergence. Seventy five BES, corresponding to 75 BACs, aligned individually to sorghum chromosomes, 10 of which having a single match amongst the sorghum chromosomes (Table 2, Class 8) while 65 had more than one possible assigned position (Table 2, Class 9).

**Table 2:** Classification of SP 80–3280 BAC end sequences as related to the alignments into the sorghum chromosomes

**BES Class**	**BES per BAC**	**Aligned BES**	**Sorghum Chromosome**	**BES Orientation**	**Distance Between BES (Kb)**	**Type**	**BAC Count**
1	2	2	Same	Opposite in (> <)	20 - 300	Concordant	42
2	2	2	Same	Same (< < or > >)	20 - 300	Discordant	1
3	2	2	Same	Opposite in (> <)	> 300	Discordant	16
4	2	2	Same	Same (<< or >>)	> 300	Discordant	12
5	2	2	Same	Opposite out (< >)	> 300	Discordant	15
6	2	2	Different	N/A	N/A	Discordant	9
7	2	1	N/A	N/A	N/A	1	10
8	2	1	N/A	N/A	N/A	> 1	65
9	2	0	N/A	N/A	N/A	N/A	22

Class 1, BAC end pairs that matched the same Sorghum chromosome at positions 20 to 300 Kb apart in opposite orientation. Class 2, BAC end pairs that matched the same Sorghum chromosome at positions 20 to 300 Kb apart in the same orientation. Class 3, BAC end pairs that matched the same Sorghum chromosome within a distance larger than 300Kb in the opposite in orientation. Class 4, BAC end pairs that matched the same Sorghum chromosome within a distance larger than 300Kb in the same orientation. Class 5, BAC end pairs that matched the same Sorghum chromosome within a distance larger than 300Kb in the opposite out orientation. Class 6, BAC end pairs that matched different Sorghum chromosome. Class 7, BAC end pairs for which only one sequence matched a sorghum chromosome at a single position. Class 8, BAC end pairs for which only one sequence matched sorghum chromosomes in more than one position. Class 9, BAC end pairs that didn’t match Sorghum chromosome.

### Distribution of BES into the sorghum chromosomes

**Figure 3:**
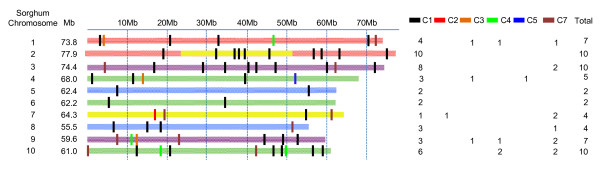
**Orthologous alignment of the BES of a random sample of 61 clones of the SP80-3280 sugarcane BAC library on the 10 sorghum chromosomes.** Sequences from the both ends of the sugarcane BAC clones were searched against the sorghum genome using BlastN, and significant hits were annotated and positioned on the corresponding sorghum chromosome. For non-repetitive sequences, positioning was based on the BAC insert size, concordance of the opposite end sequence direction and best hit. For repetitive sequences, alignment utilised only the best hit. The coloured solid lines represent the sorghum chromosomes with their predicted segmental duplication [1]. The coloured rectangles represent the sugarcane BES classes. C1, C2, C3, C4, C5, C6 and C7 refer to BESs classes as defined in Table 2. C8 and C9 classes are not represented in the figure.

A total of 112 BES, corresponding to one or both ends of 61 BACs, aligned into the 10 sorghum chromosomes (Figure 3). Eighty four BES corresponding to paired ends of 42 BACs aligned in a concordant manner. Ten BACs had only one BES aligned in a single position into a sorghum chromosome (Table 2, Class7). The other 18 BES from 9 BACs aligned in a discordant manner (Table 2 Class 2, 3, 4 and 5). The 61 BACs had their BES randomly aligned along the 10 sorghum chromosomes (Figure 3). However, chromosomes 5 and 6 presented long regions without aligned BES sequences. This could be attributed to several different factors, including bias in the constructed BAC library and regions of chromosome 5 and 6 without representation in the sugarcane genome due to sequence loss after the sugarcane/sorghum divergence. Another likely reason for the smaller number of aligned BESs on chromosomes 5 and 6 is that both of these chromosomes are richer in repetitive elements (Table 3). Since we did not align BES ends representing repetitive elements, this has introduced a bias in the BES distribution analysis. Of the 112 BESs analysed (Table 2, Class 1 to 8) only 84 (Table 2, Class 1) aligned in a concordant syntenic manner. The other 28 BESs (Class 2 to 8) aligned in a discordant manner, or each end aligned at different chromosome. This result suggests that the sugarcane genome has undergone extensive rearrangement, including inversions and translocations, relative to the sorghum genome. A sample of the concordant syntenic BACs for which insert size was estimated by restriction enzyme digestion was used to illustrate the expansions and contractions of the sugarcane genome relative to the sorghum genome (Additional file 2: Table S2). Of the 42 concordant BAC end sequence pairs, 22 aligned to syntenic regions of the sorghum genome that were larger than the estimated size of the sugarcane BAC. Other syntenic regions of the sorghum genome showed contractions compared to the corresponding sugarcane BAC (Addtional file 3: Table S 3). However, the sum of the nucleotides of the expanded and contracted syntenic regions showed a positive expansion of the sorghum genome compared to the corresponding sugarcane BACs. The sorghum syntenic regions were 29% expanded relative to the same region represented by the sugarcane BACs. This result is in keeping with the suggestion that the sugarcane genome may be 20% to 30% smaller than the sorghum genome [17]. We also investigated the nature of the genic sorghum region of conserved concordant syntenic regions relative to the sugarcane BACs (Table 3). Sorghum chromosomes (1, 2 and 3) with higher gene density and lower repetitive element content were associated with a higher proportion of sugarcane syntenic BACs. Sorghum chromosomes (5, 6 and 7) with higher repetitive content and lower gene density exhibited the lowest proportion of syntenic BACs found. These findings further suggest that the most syntenic chromosome is chromosome 2, which also shows the most expanded sequence compared to sugarcane. Additionally, the genes present in the syntenic regions of sorghum chromosomes were classified according to Gene Ontology (GO) functional categories (Additonal file 3: Table S 3). Most of the GO terms (55%) associated with the expanded sorghum regions were related to binding metabolic processes. Genes in the biosynthetic process (28%) and nitrogen compound metabolic process (24%) categories, which fall under the biological process category, were the most represented in the expanded regions. GO terms related to transferase activity (48%) were the most widely observed in the regions that were contracted in sorghum in relation to sugarcane. The most (50%) represented biological process category found in these regions was the cellular metabolic process category. Neither the contracted nor the expanded regions appeared to be significantly discrepant, in terms of GO functional categories as compared to the complete genome.

**Table 3:** Difference of expanded and contracted sorghum regions syntenic to sugarcane BACs and gene and repetitive elements content of the expanded/contracted region of sorghum

**Chromosome**	**Number of syntenic regions**	**Sum of sugarcane BAC nucleotides (bp)**	**Sum of nucleotides of syntenic sorghum regions (bp)**	**Nucleotide difference between sorghum and sugarcane syntenic regions (bp)**	**Gene density of the sorghum chromosomes (%)**	**Repetitive elements of the sorghum chromosomes (%)**	**Distribution of sugarcane BACs among sorghum chromosomes (%)**
Chromosome 1					21.3	43.4	11.5
Expanded regions	2	228,757	438,099	209,342			
Contracted regions	2	246,930	170,629	−76,301			
Chromosome 2					15.1	60.1	16.4
Expanded regions	7	843,489	1,530,484	686,995			
Contracted regions	3	390,747	307,693	−83,054			
Chromosome 3					16.8	58.2	16.4
Expanded regions	3	300,722	649,695	348,973	2.3	12.7	
Contracted regions	5	623,310	501,139	−122,171	16.9	6.5	
Chromosome 4					14.9	56.2	8.2
Expanded regions	2	228,311	542,706	314,395	5.5	15.6	
Contracted regions	1	129,840	60,597	−69,243	28.0	20.0	
Chromosome 5					8.1	65.9	3.3
Expanded regions	1	105,911	192,480	86,569	19.6	59.2	
Contracted regions	1	188,640	93,686	−94,954	13.6	47.8	
Chromosome 6					12.8	66.3	3.3
Expanded regions	0	na	na	na	na	na	
Contracted regions	2	247,660	68,939	−178,721	0.0	35.9	
Chromosome 7					9.2	66.2	6.6
Expanded regions	1	44,210	296,520	252,310	6.0	57.8	
Contracted regions	0	na	na	na	na	na	
Chromosome 8					9.0	65.6	6.6
Expanded regions	2	266,020	341,742	75,722	0.2	33.2	
Contracted regions	1	125,996	124,114	−1,882	13.1	52.1	
Chromosome 9					11.8	61.6	11.5
Expanded regions	2	221,580	423,004	201,424	27.2	23.5	
Contracted regions	1	114,955	91,453	−23,502	23.5	25.2	
Chromosome 10					12.0	60.9	16.4
Expanded regions	2	184,310	374,313	190,003	8.0	13.9	
Contracted regions	4	502,882	283,520	−219,362	12.2	5.7	
Total		4994270	6490813	1496543			

Number of regions: concordant syntenic regions with either increased or decreased size in sorghum compared to sugarcane.

Sum of sugarcane BAC nucleotides: size of sugarcane BAC in nucleotides as determined by *Not*I restriction digestion analysis.

Sum of nucleotides corresponding to syntenic sorghum regions: sum of nucleotides of the sorghum region corresponding to syntenic sugarcane BACs.

Nucleotide difference between sorghum and sugarcane syntenic regions: positive values indicate regions that are expanded and negative values indicated regions that are contracted in sorghum as related to sugarcane BACs.

Gene density of the sorghum chromosomes: percentage of gene encoding sequences (bp) in each sorghum chromosome.

Repetitive elements of the sorghum chromosomes: percentage of nucleotides corresponding to repetitive elements in each sorghum chromosome. Distribution of sugarcane BACs among sorghum chromosomes: percentage of concordant syntenic sugarcane BACs positioned in each one of the sorghum chromosome.

### Repetitive elements content

Among the total number of nucleotides of the 378 BESs analysed, 45.2% produced significant hits with sequences in repetitive elements databases (Table 4). This is in keeping with the proportion of repetitive elements observed in the sample of 19 BACs sequenced from the R570 BAC library [17]. However, repetitive elements are highly lineage-specific, and because the limited sugarcane entries in repbase the data based on BESs may be underestimated. Nevertheless, this preliminary estimation suggests that the repetitive element counterpart of the sugarcane genome, may be smaller than that of the sorghum genome, which contains 61% repetitive sequences, most of which are located in centromeric and pericentromeric regions [14]. Most (98%) of the repetitive nucleotides found in the BES reads corresponded to transposable elements; 85.2% were LTR retrotransposons, of which 48.1% were assigned to the Copia family and 51.6% to the Gypsy family. Non-LTR retrotransposons of the L1, RTE, SINE and SINE/tRNA families corresponded to 3.5% of the total repetitive element nucleotides (Table 4). DNA transposable elements belonging to the EnSpm, Harbinger, Helitron, MuDr and hAT families represented 10.9% of the total BES repetitive nucleotides. Few sequences were found to correspond to integrated viruses (0.8%) or simple repeats (1.2%).

**Table 4:** Summary of repetitive sequences among the sugarcane BESs

**Repeat Element**	**Number of elements**	**Length (bp)**	**% of Total Bases**
Transposable Element	293	160624	44.29
RNA transposon	234	142693	39.35
LTR Retrotransposon	221	136899	37.76
Copia	96	65873	18.17
Gypsy	123	70697	19.50
Non-LTR Retrotransposon	13	5794	1.59
L1	7	2626	0.72
RTE	4	2943	0.81
SINE	2	225	0.06
SINE2/tRNA	2	225	0.06
DNA transposon	59	17931	4.94
EnSpm	13	5358	1.48
Harbinger	11	2728	0.75
Helitron	2	1197	0.33
MuDr	6	2515	0.69
hAT	9	3111	0.86
Integrated Virus	2	1231	0.34
Caulimoviridae	2	1231	0.34
Simple Repeat	3	1923	0.53
Satellite	3	1923	0.53
Total	298	163778	45.16

## Discussion

Two BAC libraries from the Reunion Island sugarcane cultivar R570, one constructed with DNA isolated from the commercial variety [23] and, the other constructed with DNA isolated from selfed progenies of R570 [24] are current available. These libraries have contributed with BAC sequencing for various purposes. Here, we described the construction and initial analyses of a new sugarcane BAC library prepared with genomic DNA from a Brazilian elite commercial sugarcane variety. This BAC library exhibits genome coverage of 5–6 times the monoploid chromosome set of sugarcane. The genome coverage was estimated based on a size of 760–930 Mb for the monoploid sugarcane genome [7]. However, in a previous study, syntenic alignment of 19 sugarcane BAC sequences from the R570 BAC library into the 20 sorghum chromosome arms revealed predominant local DNA sequence expansion of the sorghum genome in the regions syntenic with the sugarcane BAC sequences [17]. These results suggested that the monoploid sugarcane genome could be 20% smaller than the 730 Mb sorghum genome. The alignment of the 42 BES pairs into concordant syntenic regions of the sorghum genome revealed 29% expansion of sorghum in relation to the sugarcane genome. This result is in keeping with the results observed for the R570 BAC library and suggests that the size of the monoploid sugarcane genome could be on the order of 580 Mb. If this is correct, the coverage of the SS_SBa BAC library could be on the order of 8 times the sugarcane monoploid genome.

The use of the sorghum genome sequence as a template to assemble the sugarcane genome has been proposed based on the close similarity between the two species [25,26]. The sequence of BAC clones from the R570 BAC library and comparison of its gene and repetitive element content to that of sorghum improved confidentiality with respect to these assumptions [16,17]. Sequence analysis of 19 BAC from the R570 BAC library revealed that almost 85% of its gene-encoding sequences are syntenic with sorghum orthologs [17]. We analysed the sorghum chromosomes for gene density as related to the distribution of the SP80-3280 BES. Sorghum chromosomes 1, 2 and 3 showed the highest gene density and had increased number of aligned sugarcane BESs (Table 3). Chromosomes 5 and 6 has reduced gene density were richer in repetitive elements and showed fewer aligned sugarcane BESs (Table 3).

The library described in this report is from an elite commercial sugarcane variety that has been cultivated on hundreds of thousands of hectares in a range of different environments, including regions of less favourable soils in terms of water and nutrient availability. This library would be useful in providing additional information regarding the allelic composition selected by breeders. The overlapping BACs in this library may represent different homeologous chromosomes from both *S. officinarum* and *S. spontaneum* parents. Since *S. officinarum* contributes mainly with yield and sugar alleles and, *S. spontaneum* contributes mainly with stress tolerance genes, the sequences of overlapping BACs representing both species could be identified by high stringency filter hybridisation with DNA from the two parents [16]. Furthermore, their gene and allele content could be identified, and the contribution of each of the parental genes to disease resistance and sugar content could be assigned. Additionally, expression patterns obtained using next generation platforms could provide additional useful information regarding this valuable genetic resource.

## Conclusions

Sugarcane is a main crop for both sugar and bioenergy generation. To address the projections for sugarcane production, breeding and biotechnology approaches must be developed in the next few years, to assist the selection of high sugar yield varieties adapted to tropical and sub-tropical regions. Sequencing the genome of this complex crop may help to identify agronomically useful genes, either per se or through comparative genomics, and could also assist in the development of biotechnology tools for sugarcane improvement. This report describes the construction and preliminary analyses of a sugarcane BAC library from DNA isolated from a Brazilian elite sugarcane variety. The library comprises large insert clones and possesses 5–6 times coverage of the monoploid sugarcane genome. Sequencing and alignment of BAC end sequences from a sample of this library into orthologous regions of the sorghum genome revealed that the library presents sound genome coverage. In addition, comparison of the syntenic regions of the sorghum genome with respect to BAC end sequence pairs confirmed that the sugarcane genome might be between 20% and 30% smaller than the sorghum genome. This library represents a new resource for the community interested in sugarcane breeding and biotechnology coupled with sustainable bioenergy generation.

## Methods

### Germplasm and plant tissue processing

Twenty 10-week-old, field-grown sugarcane plants of the SP80-3280 variety were generously provided by the Cosan company (http://www.cosan.com.br). The plants were harvested at Usina Santa Helena in Fazenda Santo Antonio (GPS coordinates −22.735657, -47.305069), Piracicaba, State of São Paulo, Brazil. The plants were subjected to a 30-hour dark treatment, after which the healthy young leaves were collected, quickly washed to remove debris and immediately frozen by submersion in liquid nitrogen. The frozen leaves were stored at −80°C until use.

### Preparation of high molecular weight (HMW) sugarcane DNA in agarose plugs

The sugarcane SP-803280 BAC library was constructed in the Arizona Genomics Institute (AGI) using standard protocols [27,28]. Fifty grams of frozen tissue were ground under liquid nitrogen with a mortar and pestle. The ground tissue was transferred to a 1-L Erlenmeyer flask containing 500 mL of pre-chilled extraction buffer (10 mM Tris–HCl, pH 8.0, 10 mM EDTA, pH 8.0, 100 mM KCl, 0.5 M sucrose, 4 mM spermidine, 1 mM spermine, 2.0% w/v PVP-40, 0.13% w/v sodium diethyldithiocarbamate trihydrate and 800 μl β-mercaptoethanol). The suspension was gently shaken for 15 min, and the homogenate was filtered into an Erlenmeyer flask containing 500 mL of pre-chilled extraction buffer with 1.7% Triton X-100. The suspension was kept on ice for 15 min and then centrifuged for 15 min at 3,250 rpm at 4°C. The resulting pellet was resuspended in pre-chilled extraction buffer, incubated for 5 min in a water bath at 45°C and gently mixed with 1/3 v/v of 1.0% low melting temperature agarose that was previously prepared in extraction buffer and held at 45°C. The mixture was transferred to plug moulds and allowed to solidify. Forty-six plugs were transferred into a 50-mL Falcon tube containing 40 mL of proteinase K solution (0.5 M EDTA pH 9.2, 1.0%N-lauroylsarcosine, 40 mg proteinase K and 2% PVP), and the tube was incubated in a hybridisation oven at 50°C with gentle rotation for 24 h. The plugs were then washed with fresh proteinase K solution for an additional 24 h. Subsequently, the plugs were washed five times for 1 h at room temperature using 40 mL T10E10 containing phenylmethylsulfonyl fluoride (PMSF; 10 mM Tris–HCl, 10 mM EDTA, 1 mM PMSF, pH 8.0) and five times for 1 h with T10E1 plus PMSF (10 mM Tris–HCl, 1 mM EDTA, 1 mM PMSF, pH 8.00). The plugs were stored in TE at 4°C.

### Restriction digestion of HMW DNA and isolation of size-selected fragments

Eight DNA plugs were partially digested for 20 minutes with 0.6 U of the *Hind*III restriction enzyme for each half plug. The digested samples were loaded into a 1.0% agarose gel and subjected to pulsed-field gel electrophoresis (PFGE). DNA was visualised using a UV transilluminator, and fragments containing DNA ranging from 90 to 450 Kb were cut from the gel slabs. The fragments were subsequently purified through second and third PFGE runs to remove small trapped DNA fragments [27]. The gel fractions containing sized fragments were recovered from the gel slabs and stored at 4°C.

### Ligation of sized DNA fragments

High-molecular-weight genomic DNA fragments (120–200 ng) were ligated into a HindIII- linearized and dephosphorylated pAGIBAC1 plasmid vector [27]. The ligation reactions were incubated in a water bath at 16°C for 19 h, transferred to 0.1 M glucose/1.0% agarose and allowed to desalt for 1.5 h on ice. The ligations were transferred into new microcentrifuge tubes and stored at 4°C. The ligation samples were tested to determine the transformation efficiency and cloned insert quality. For the final transformations, 2.0 μl of ligation mixture was used to electroporate 20 μl of DH10B T1 phage-resistant E. coli cells (Invitrogen). The transformed cells were transferred into 3 mL of SOC media and incubated at 37°C for 1 h in a shaker at 250 rpm, followed by the addition of an equal volume of sterile glycerol and gentle shaking for 3 min, after which the mixtures were immediately frozen by submersion into liquid nitrogen and stored at −80°C. Subsequently, the cells were thawed and plated on 22.5 x 22.5 cm plates containing solid LB medium with 12.5 μg/mL chloramphenicol, 80 μg/mL X-gal and 100 μg/mL IPTG. The plates were incubated at 37°C overnight. White recombinant colonies were transferred into liquid LB medium containing 12.5 mg/mL chloramphenicol and incubated overnight at 37°C. The transformed E. coli from ligations that contained large inserts were arrayed into 96 x 384-well plates to constitute the SS_SBa BAC library.

### Quality control and BES sequencing and analysis

Two 96-wells plates were set up using two clones from each 384-well plate of the SS_SBa BAC library. BAC DNA was isolated from these two 96-well plates, digested with NotI and separated by PFGE for fragment sizing. DNA from the same 192 BAC clones was used for BAC end sequencing with an ABI 3730 sequencer at the AGI facility. The BESs were trimmed for vector and low quality sequences using the SUCEST project trimming procedure [29]. The trimmed sequences were compared to the NCBI GenBank non-redundant protein database using BlastX (E-value cutoff of 1e-5), to NCBI GenBank nucleotide database, to sorghum, maize and rice genome sequences, sugarcane ESTs and BAC sequences and to the sugarcane chloroplast genome [21] and rice mitochondria genome [22] using BlastN. For all BlastN searches, an E-value cutoff of 1e-20 was used. Additionally, for chloroplast and mitochondria BlastN searches a cutoff of 80% coverage was used. Repeats in the sugarcane BES were masked [30] and identified through searches for similarity to grass sequences in the RepBase [31] with Censor [32]. The BES sequences have been submitted GenBank/NCBI under ID: (dbGSS JS672894 - JS673271).

### Comparative analysis and alignment of BESs into the sorghum genome

Regions of microsynteny between sorghum and sugarcane were mapped by the alignment of BESs onto sorghum genome sequences using BlastN alignments with an E-value cutoff of 1e-20. A BES was considered microsyntenic if both ends mapped within 20 Kb and 300 Kb in the opposite orientation. When the two ends were opposite oriented one to another, the region was considered collinear [33,34]. Otherwise, the region was considered to be rearranged between the two species. The best score sum of two ends was used to select among multiple mapping possibilities. Gene density and Gene Ontology analyses of the sorghum chromosomes and syntenic regions were based on Phytozome (V7.0) and the JGI sorghum genome annotation. Repetitive elements in the sorghum chromosomes and syntenic regions were identified with Censor [32] using RepBase [31].

## Competing interests

The authors declare that they have no competing interests

## Authors' contributions

TRSF participated in sample collection, DNA preparation, data analysis and help drafting of the manuscript. TRSF and JT constructed the sugarcane BAC library under the supervision of DK, JSSA and RW. MJS was involved in sampling and coordination. VO and FRS performed the bioinformatics analysis, and PA directed the strategy of the work toward sequencing the sugarcane genome using a BAC library and drafted the manuscript. All of the authors have read and approved the final manuscript.

## Supplementary Material

Additional file 1 **Table S1.** Sequence ID, annotation and orthologous positioning of 384 BES from 192 clones of the Sugarcane SP 80**-**3280 BAC Library into sorghum chromosomes. Orthologous sequence present in the maize and rice genomes are also displayed. Click here for file

Additional file 2 **Table S2.** Position coordinates of 42 BES pairs into the 10 sorghum chromosomes. Click here for file

Additional file 3 **Table S3.** Gene ontology (GO) categories for genes in the expanded and contracted regions of the color genome compared with the complete genome. Click here for file
